# Model‐driven design of a minimal medium for *Akkermansia muciniphila* confirms mucus adaptation

**DOI:** 10.1111/1751-7915.13033

**Published:** 2018-01-26

**Authors:** Kees C. H. van der Ark, Steven Aalvink, Maria Suarez‐Diez, Peter J. Schaap, Willem M. de Vos, Clara Belzer

**Affiliations:** ^1^ Laboratory of Microbiology Wageningen University & Research Stippeneng 4 6708 WE Wageningen The Netherlands; ^2^ Laboratory of Systems and Synthetic Biology Wageningen University & Research Stippeneng 4 6708 WE Wageningen The Netherlands; ^3^ Department of Bacteriology and Immunology RPU Immunobiology University of Helsinki Haartmanikatu 4 002940 Helsinki Finland

## Abstract

The abundance of the human intestinal symbiont *Akkermansia muciniphila* has found to be inversely correlated with several diseases, including metabolic syndrome and obesity. *A. muciniphila* is known to use mucin as sole carbon and nitrogen source. To study the physiology and the potential for therapeutic applications of this bacterium, we designed a defined minimal medium. The composition of the medium was based on the genome‐scale metabolic model of *A. muciniphila* and the composition of mucin. Our results indicate that *A. muciniphila* does not code for GlmS, the enzyme that mediates the conversion of fructose‐6‐phosphate (Fru6P) to glucosamine‐6‐phosphate (GlcN6P), which is essential in peptidoglycan formation. The only annotated enzyme that could mediate this conversion is Amuc‐NagB on locus Amuc_1822. We found that Amuc‐NagB was unable to form GlcN6P from Fru6P at physiological conditions, while it efficiently catalyzed the reverse reaction. To overcome this inability, *N*‐acetylglucosamine needs to be present in the medium for *A. muciniphila* growth. With these findings, the genome‐scale metabolic model was updated and used to accurately predict growth of *A. muciniphila* on synthetic media. The finding that *A. muciniphila* has a necessity for GlcNAc, which is present in mucin further prompts the adaptation to its mucosal niche.

## Introduction

*Akkermansia muciniphila* is a mucin‐degrading bacterium that is present in the intestinal tract of a majority of people (Derrien *et al*., 2008). *Akkermansia muciniphila* was first isolated using purified hog gastric mucus as sole carbon and nitrogen source (Derrien *et al*., 2004). In addition to the degradation of intestinal mucins, *A. muciniphila* was shown to be closely associated to colonic epithelial cells producing these mucins (Derrien *et al*., 2011). The adaptation to this niche is exemplified by the capabilities of *A. muciniphila* to utilize low concentrations of oxygen present in the mucus layer (Ouwerkerk *et al*., 2016), even though it was previously characterized as a strictly anaerobic bacterium (Derrien *et al*., 2004 (Derrien *et al*., 2004). The efficient use of mucin by *A. muciniphila* was shown in an *in vitro* intestinal model and upon addition of mucus, *A. muciniphila* abundance showed over 10 000‐fold increase, the highest ever observed in this model (Van Herreweghen *et al*., 2017).

The mucin in the mucus layer lining the intestinal track is composed of a peptide backbone abundantly decorated with O‐linked glycans (Johansson *et al*., 2011; Thomsson *et al*., 2012). The peptide backbone is rich in threonine, serine, cysteine and proline, while the glycans are composed of a plethora of sugar groups that contain mannose, galactose, fucose and *N*‐acetylhexosamines such as *N*‐acetylglucosamine (GlcNAc), and *N*‐acetylgalactosamine (GalNAc). *Akkermansia muciniphila* is capable of fermenting some of these sugars including galactose, fucose, glucose, GlcNAc and GalNAc, which have been reported to be used for both energy generation and as carbon source (Desai *et al*., 2016; Ottman *et al*., 2017a). However, the degradation of these sugars is only possible in the presence of mucin or large amounts of a tryptic digest of casein (Desai *et al*., 2016; Ottman *et al*., 2017a).

The genome‐encoded metabolic potential of *A. muciniphila* has been previously exploited to design a genome‐scale model of its metabolism (van Passel *et al*., 2011; Ottman *et al*., 2017a). Extensive model curation and evaluation led to the prediction of an auxotrophy for l‐threonine as well as predictions regarding the production of the short‐chain fatty acids (SCFA) acetate and propionate, and succinate in the absence of vitamin B12. The production of these SCFAs was confirmed by growth experiments, however, in deviating ratios compared with the predictions. The addition of l‐threonine to the growth media did increase growth, but its essentiality for growth was not confirmed due to the presence of the partially undefined casein hydrolysate in the medium (Ottman *et al*., 2017a).

Inverse correlations have been reported between the relative abundance of *A. muciniphila* and diseases and disorders as reviewed previously (Derrien *et al*., 2016). Moreover, in a series of preclinical interventions in mice, it was shown that *A. muciniphila* reversed diet‐induced metabolic fat‐mass gain and insulin resistance (Everard *et al*., 2013; Plovier *et al*., 2017) possibly due to outer membrane produced pili (Ottman *et al*., 2017b; Plovier *et al*., 2017). To better understand the physiological properties, clinical effects and function of *A. muciniphila* (Ottman *et al*., in press), it is essential to have a defined minimal medium for growth. Such a medium can also be the basis for the application of *A. muciniphila* as a therapeutic microbe as the medium components should be defined and preferably of non‐animal origin before clinical tests can be conducted. Here, we present a completely defined and minimal medium that supports growth of this beneficial microbe. We confirmed that the amino acid l‐threonine is essential for growth and observed that the addition of either GlcNAc or GalNAc was essential for the growth of *A. muciniphila*. Furthermore, we discovered *A. muciniphila* does not code for a functional GlmS and hence requires the exogenously added GlcNAc or GalNAc not only for fermentation, but also for peptidoglycan formation.

## Results and discussion

### GlcNAc is essential for *A. muciniphila* growth

**Figure 1:**
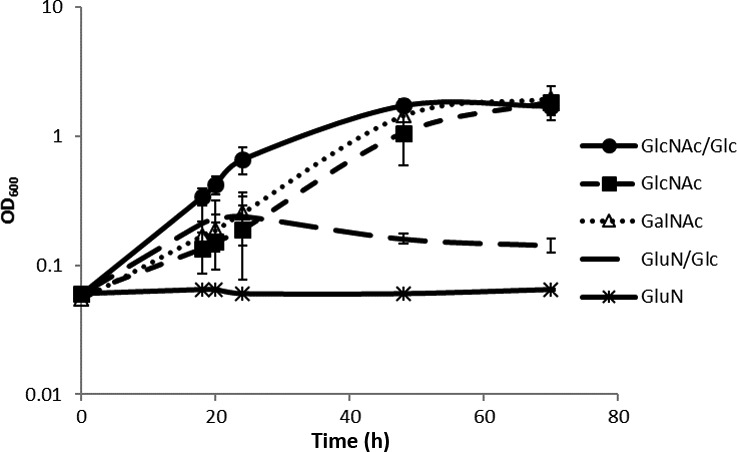
Growth of *Akkermansia muciniphila* on CP medium supplemented with l‐threonine and different sugars. All sugars were supplemented to a total of 25 mM. The negative control was supplemented with GlcNAc/glucose and not inoculated. The data shown are averages of three biological replicates and two technical duplicates for each. The experiment was performed twice in duplicate; bars indicate standard deviations.

*Akkermansia muciniphila* was isolated using porcine gastric mucus, and it was shown that this complex substrate could serve as the sole nitrogen and carbon source (Derrien *et al*., 2004). Limited growth was also observed when large amounts of tryptone, a tryptic digest of casein, were added as nitrogen source in combination with monosaccharides (Ottman *et al*., 2017a). This can be due to the presence of either amino acids or glycans in both complex substrates. Hence, the dependency of *A. muciniphila* on exogenous nitrogen‐containing compounds and sugars was tested in a series of growth experiments (Fig. 1). The amino sugars tested (GlcNac, GalNac and GlcN) in these experiments were selected based on their presence in mucin glycans. We tested the essentiality of l‐threonine by omitting it in the medium in the presence of ammonium and other amino acids, which resulted in no growth (Fig. S1). This amino acid was selected based on the *A. muciniphila* genome‐scale constraint‐based model of metabolism, referred to as ‘the model’ hereafter (Ottman *et al*., 2017a). The final composition of the minimal medium is a basal mineral medium, called CP medium (Derrien *et al*., 2004; Plugge, 2005), containing per litre 0.4 g KH_2_PO_4_; 0.53 g Na_2_HPO_4_; 0.3 g NaCl; 0.1 g MgCl_2_ 6(H_2_O); 0.11 g CaCl_2_; 1 ml alkaline trace element solution; 1 ml acid trace element solution; 1 ml vitamin solution; 0.5 mg resazurin; 4 g NaHCO_3_; 0.25 g Na 2S.7–9H 2O as described before, but without ammonium chloride (Derrien *et al*., 2004) and added l‐threonine and GlcNAc or GalNAc. The composition of trace elements and vitamins was used as described before (Plugge, 2005).

A specific growth rate of 0.056 h^−1^ was observed for medium containing 25 mM GlcNAc, and a value of 0.084 h^−1^ was found for growth on 25 mM GalNAc (Table 1), which is no significant difference (*P* > 0.05). The specific growth rate was increased from 0.056 h^−1^ to 0.122 h^−1^ when half of the GlcNAc was replaced by an equimolar amount of glucose, which is a significant increase (*P* < 0.05). The final density of the cells is similar for growth on GlcNAc, GalNAc and GlcNAc/glucose (Fig. 1). When GlcN was used as substrate, growth was only observed with the addition of glucose, at a very low specific growth rate of 0.005 h^−1^.

**Table 1:** Specific growth rate per hour (μ) of *Akkermansia muciniphila* on different sugars, with 6** **g/l l‐Thr

Sugars	μ (h^−1^)	SD
GlcNAc/Glc	0.122	0.036
GlcNAc	0.056	0.023
GalNAc	0.084	0.014
GlcN/Glc	0.005	0.004
GlcN	−0.001	0.002
Fru	0.000	0.000
Glc	0.000	0.000
Neg. control	0.000	0.000

**Figure 2:**
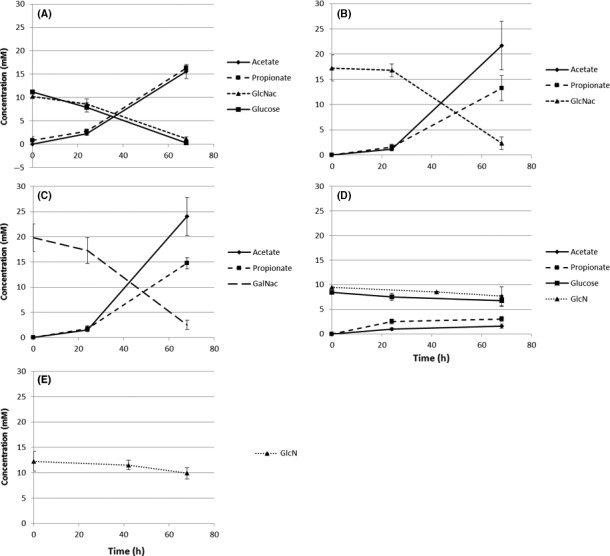
SCFA production and degradation of sugars. The degradation of GlcNAc and glucose results in a 1:1 ratio of acetate and propionate (A). The degradation of GlcNAc and GalNAc results in similar acetate and propionate ratios of 1.5:1 to 2:1 (B, C). The production of acetate and propionate on glucose and GlcN is in a 1:2 ratio (D). GlcN is still degraded when not supplemented with glucose, but no SCFA production was observed (E). The experiment was performed twice in duplicate; bars indicate standard deviations.

The consumption and production of metabolites by *A. muciniphila* after growth was measured by HPLC and used to obtain insight into the pathways operating (Fig. 2), which can be used to study the degradation pathways of sugars. This analysis showed that the consumption rates of glucose and GlcNAc were the same when both were present in the growth medium (Fig. 2A). The growth differences observed (Fig. 1) could be influenced by transcriptional adaptations to the presence of amino sugars. The sugar degradation profiles (Fig. 2A and D) indicate that glucose is consumed at a similar rate throughout the analysed time‐course, indicating that no additional adaptations are required for sugar consumption. In case of GlcNAc and GalNAc degradation by *A. muciniphila*, the ratio of acetate and propionate production was the same, between 1.5:1 and 2:1 (Fig. 2B and C). Combination of GlcNAc and glucose in the medium leads to a 1:1 ratio (Fig. 2A). Degradation of glucose in the presence of GlcN produced acetate and propionate in a 1:2 ratio, GlcN as a sole carbon source was not able to sustain growth, nevertheless is degraded albeit in low amounts and did not contribute to SCFA production (Fig. 2D and E). This level of SCFA production on glucose was in line with the predictions by the model (Ottman *et al*., 2017a), but not with previous findings in growth experiments where an acetate to propionate ratio of 1:1 was found (Ottman *et al*., 2017a). The accurate determination of SCFA production on monosaccharides needed the minimal medium described here as on complex and undefined media with mucus or casein hydrolysate such measurements were inaccurate.

The production of SCFA after degradation of the acetylated amino sugars GlcNAc and GalNAc indicated that these compounds are degraded for the generation of energy in addition to their possible use for the formation of peptidoglycan. A ratio of 1.5:1 to 2:1 is in line with previous findings (Ottman *et al*., 2017a). The metabolic model predicts a 5:4 ratio, which slightly lower than the measured value (Ottman *et al*., 2017a). The early stagnation of growth on GlcN and glucose while there is some production of SCFA shows that the cells are active and alive, but limited in growth. This shows again that either GlcNAc or GalNAc is essential for growth in terms of biomass formation.

**Figure 3:**
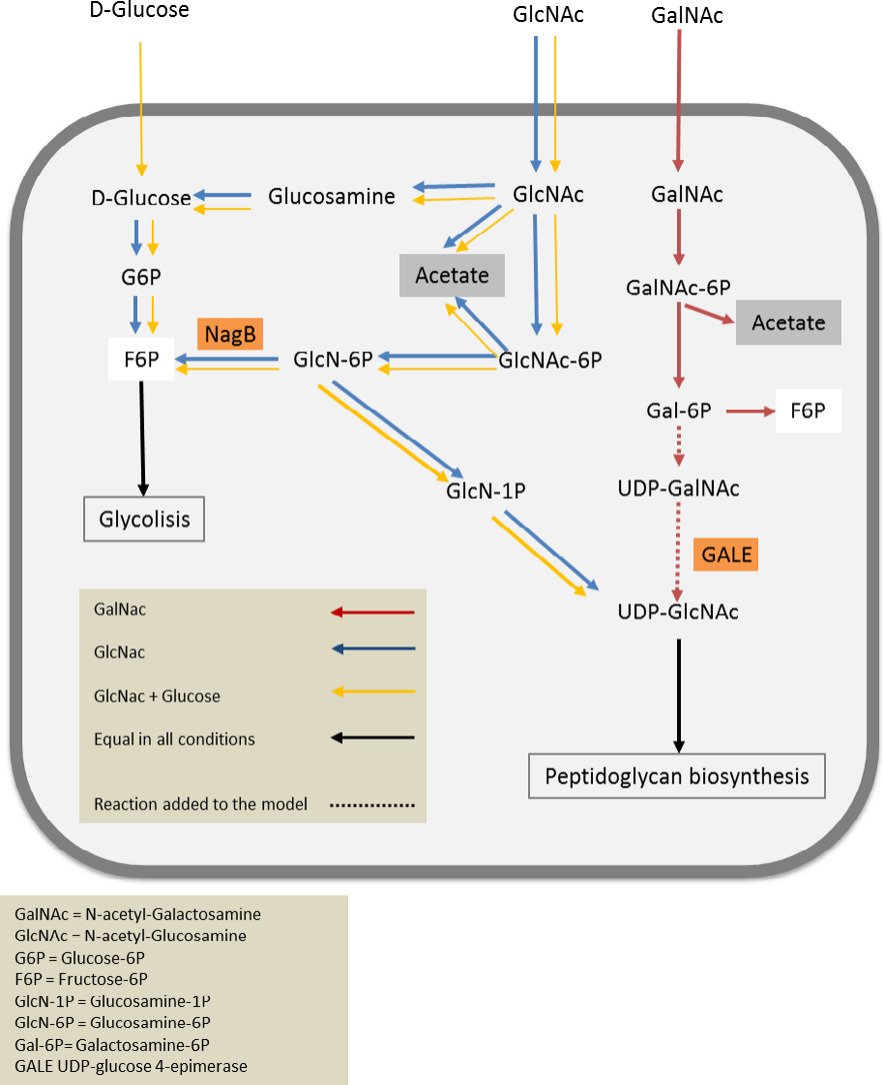
Core biochemical network of *Akkermansia muciniphila* with the distribution of expected fluxes in the main catabolic pathways under optimal growth conditions described in the article. Arrow width indicates relative changes in the fluxes on different substrates GalNac (red), GlcNac (blue) and mixture of GlcNac and glucose (yellow). Reactions for which all conditions showed similar fluxes are represented by black arrows. Model simulations were performed with equimolar total amounts of sugar. Figure has been adapted from (Ottman *et al*., 2017a).

Although it has been suggested that *A. muciniphila* is able to degrade GalNAc (Ottman *et al*., 2017a), the previous metabolic reconstruction of *A. muciniphila* contained no GalNac degradation pathways due to the current lack of reference degradation pathways in the MetaCyc database (Caspi *et al*., 2014). The growth of *A. muciniphila* on GalNAc could possibly be explained by an isomerizing step from UDP‐GalNAc to UDP‐GlcNAc, mediated by the enzyme UDP‐glucose 4‐epimerase encoded on locus Amuc_1125 (AMUC_RS06020) (Thoden *et al*., 2001; Bernatchez *et al*., 2005; van Passel *et al*., 2011). The growth of *A. muciniphila* on GlcN in combination with glucose could be due to acetylation of GlcN6P (Fig. 3). This acetate group is known to be donated by acetyl‐CoA, which can only be formed if a sugar is degraded first. Due to this constraint, the growth may be slower because it requires glucose to form acetyl‐CoA. Additionally, from the metabolic model point of view, growth on GlcN is only impaired by the lack of a transport mechanism as we could not identify a gene in the *A. muciniphila* genome that would code for a transporter for GlcN. Therefore, it could be that the transport of GlcN to the cytoplasm is limited to transportation by other sugars transporters and thereby very inefficient.

The results indicated that amino sugars (GlcNAc, GalNAc and limited on GlcN) could support growth of *A. muciniphila,* but not glucose or fructose (Table S1). This suggested that *A. muciniphila might* lack the ability to aminate fructose‐6‐phospgate (Fru6P) to form glucosamine‐6‐phosphate (GlcN6P), which is an essential step in peptidoglycan synthesis (Barreteau *et al*., 2008; Durand *et al*., 2008) (Fig. 3). This reaction is catalyzed by the enzyme GlmS (see below), which mediates the attachment of an amino group donated by glutamine to Fru6P to form GlcN6P (Durand *et al*., 2008). This phosphorylated sugar is subsequently converted to *N*‐acetylglucosamine‐6‐phosphate after the addition of an acetate group and isomerized to *N*‐acetylglucosamine‐1‐phosphate (Barreteau *et al*., 2008; Fig. 3). The enzyme GlmS is essential in all peptidoglycan‐forming bacteria (Milewski, 2002), and *Escherichia coli* glms mutants are only viable if the growth medium is supplemented with GlcNAc (Wu and Wu, 1971). This indicates that the GlcNAc can be phosphorylated and used for peptidoglycan synthesis directly by *A. muciniphila*, an *Akkermansia* GlcNac shortcut (Fig. 3).

### Genomic analysis of *A. muciniphila* for the identification of glucosamine‐6‐phosphate deaminase

We mined the *A. muciniphila* genome to identify genes encoding enzymes mediating conversions between Fru6P and GlcN6P. This lead to the finding that the genome of *A. muciniphila* does not code for a gene with high similarity to a characterized GlmS, see Table S5. The highest identity to *E. coli* GlmS was a von Willebrand factor type A domain protein (KXT50996.1) with an identity of 26% and an Expect (*E*)‐value of 0.68, indicating a very low likelihood that this protein is a GlmS homologue (Table S6) (Altschul *et al*., 1990, 1994). There are also no gene homologues to GlmS from *Verrucomicrobium spinosum*, the type strain for Verrucromicrobiae, and thus a relative of *A. muciniphila* (Table S7). Therefore, we consider *A. muciniphila* does not code for GlmS. The gene that encodes for glucosamine‐6‐phosphate deaminase (CDB55261.1) on locus amuc_1822 (AMUC_RS09725) is the only candidate gene that expresses an enzyme which could mediate the reaction between GlcN6P and Fru6P (Table S4). No homologues were found in the *A. muciniphila* genome. The analogue in *E. coli* is named NagB. The directionality of this enzyme from different organisms is ambiguous (Vincent *et al*., 2005). It has been found to predominantly mediate the reaction in the deaminating direction, but aminating activity has been found as well (Tanaka *et al*., 2005; Kwiatkowska‐Semrau *et al*., 2015). Hence, we focused our attention to the kinetic characterization of the *A. muciniphila* NagB homologue. It should be noted that the recent genome sequencing of 39 *A. muciniphila* isolates might result in the discovery of genes coding for a functional NagB homologue, especially because of the great differences found between strains in the amino sugar metabolism (Guo *et al*., 2017).

### Enzyme assays confirm the deamination properties of glucosamine‐6‐phosphate deaminase

We observed that *A. muciniphila* was not able to metabolise fructose and does not grow on glucose, while GlcN supports growth, although very limited (Table 1). To assess the capabilities of *A. muciniphila* to convert fructose‐6‐phosphate (Fru6P) into glucosamine‐6‐phosphate (GlcN6P) and vice versa, we overproduced the only possible candidate enzyme, termed Amuc‐NagB, encoded by gene amuc_1822 (AMUC_RS09725) in *E. coli* BL21 (DE3) with a C‐terminal His‐tag and purified it to apparent homogeneity as a 33‐kDa protein (Fig. S2). The purified Amuc‐NagB was assayed for conversions in both the aminating and deaminating direction and with all possible substrates involved (Table S2).

At non‐physiological and saturating conditions, we found that the enzyme Amuc‐NagB can mediate the reaction combining Fru6P and NH_4_ to produce GlcN6P, with Km values for Fru6P and NH4 of 5.5 ± 0.9 mM and 41.3 ± 12.8 mM, respectively (Table 2), as calculated based on triplicate assays. However, the Km of GlcN6P value for the deaminating direction is much lower at 2.4 ± 0.2 mM (Table 2). The addition of GlcNAc6P as possible allosteric activator had no significant influence on the Km values (*P* > 0.05). On top of this, the activity of the purified Amuc‐NagB on Fru6P with glutamine was measured to determine the possibility for this enzyme to mediate the conversion of Fru6P + glutamine □ GlcN6P + glutamate [EC 2.6.1.16]. No activity was found using these substrates. Additional controls using enzyme without substrates and substrate without enzyme also showed no activity (Table S8).

**Table 2:** Enzyme kinetics of *Akkermansia muciniphila* NagB with and without GlcNAc6P. The addition of 0.25** **mM GlcNAc6P as activator has no influence on the kinetics of the enzyme

Activator: GlcNAc6P (mM)	Km (mM)		*P*‐value
	0 (*n* = 3)	25 (*n* = 2)	
NH4	41.3 ± 12.8	50.6 ± 10.5	0.54
Fru6P	5.5 ± 0.9	8.4 ± 2.7	0.48
GlcN6P	2.4 ± 0.2	2.1 ± 0.8	0.75

The Km value of 41.3 mM for NH_4_ is high, but ammonium is not toxic at this concentration (Fig. S3), and these concentrations are described to be present in the gut (Macfarlane *et al*., 1986, 1992). The ammonium present in the gut diffuses through the cell to almost equilibrium (Muller *et al*., 2006), so the required ammonium concentration for the aminating direction could take place as such in the *A. muciniphila* cells. However, the Km value of Fru6P at 5.5 mM is rather high, this compound is described to be toxic in these amounts in many bacteria (Kadner *et al*., 1992). Additionally, the Km value of GlcN6P is lower at 2.4 mM. GlcN6P is the only compound involved in the deaminating direction (Table 2, Table S8).

The kinetic values found for Amuc‐NagB are in line with previously characterized NagB‐like enzymes (Calcagno *et al*., 1984), except for the absence of the need for an allosteric activator. In all cases, GlcNAc6P is described as an allosteric activator (Alvarez‐Anorve *et al*., 2005, 2016). Our results clearly show that the overexpressed Amuc‐NagB is not allosterically activated by GlcNAc6P (Table 2).

Considering the kinetic values of Amuc‐NagB, it is unlikely for the aminating reaction to occur at physiological conditions, as also postulated before (Calcagno *et al*., 1984). We assume an alternative route to synthesize phosphorylated amino glucoses (GlcN6P, GlcN1P, GlcNAc6P and GlcNAc1P) is taking place, which is provided by the addition of non‐phosphorylated GlcNAc or GalNAC to the growth medium. We propose that GlcNAc can be used by *A. muciniphila* to form peptidoglycan intermediates and ultimately peptidoglycan via the *Akkermansia GlcNac* shortcut (Fig. 3).

### Optimization of the *A. muciniphila* genome‐scale constraint‐based model of metabolism

The growth on different sugars was compared with predictions from the *A. muciniphila* model as presented before (Ottman *et al*., 2017a). The initial version of the model (AkkMuc_588 v1) predicted growth on all sugars testes, except GlcN, as discussed above. This is in discrepancy with growth in minimal medium and can be explained by the absence of GlmS that was assumed to be present in the model due to the use of gap‐filling algorithms (Table 3). Based on these findings, the model was adjusted by deleting the reaction Fru6P + Glutamine □ GlcN6P + Glutamate [EC 2.6.1.16]. This yielded correct growth predictions for glucose and GlcNAc. The adjusted model (AkkMuc_588 v2) still did not predict growth on GalNAc. This was solved by the additional annotation of UDP‐glucose 4‐epimerase on locus Amuc_1125 (AMUC_RS06020) as UDP‐GlcNAc 4‐epimerase and which supports the formation of UDP‐GalNAc from GalNAc as discussed above. The epimerase has previously shown to bidirectional mediate the conversion of UDP‐glucose to UDP‐galactose and UDP‐GlcNAc to UDP‐GalNAc in both bacteria and human (Thoden *et al*., 2001; Bernatchez *et al*., 2005). The improved version of the model contains 748 reactions describing conversions among 737 metabolites and can be found in SBML and table (xls) formats in the Supplementary Information.

**Table 3:** Growth comparison between *in vitro* and *in silico* analyses

Sugar 1	Sugar 2	AkkMuc_588 v1 (y/n)	AkkMuc_588 v2 (y/n)	AkkMuc_588 v2 (au)	Lab (μ) (h^−1^)
Glucose	GlcN	y	n	0	0.005
GlcN		n	n	0	0
GlcNAc		y	y	0.09	0.056
GlcNAc	Glucose	y	y	0.13	0.122
GalNAc		y	y	0.09	0.084
Glucose		y	n	0	0

Qualitative predictions by the adjusted model (AkkMuc_588 v2) showed higher similarity to our laboratory experiments than the original model (Table 3). The only small difference between the model and laboratory experiments is the growth on a medium containing GlcN and glucose. While the model predicts no growth, a specific growth rate of 0.005 h^−1^ was observed in the laboratory experiment. This could be caused by a limited uptake of GlcN, even though there is no GlcN transporter annotated. Simulations with an *in silico* generated mutant with a hypothetical GlcN transporter show that the absence of this transporter is the sole reason for the lack of growth predicted for this sugar. This small uptake could explain the low growth rate in the presence of both GlcN and glucose, thereby providing full agreement between the model qualitative predictions and the experimental results. Nevertheless, the role of glucose remains unclear, as from the experiments, we see that it would be essential to enable this possible low uptake rate.

Concluding, we have shown that GlcNAc or Galnac with the addition of l‐threonine are essential for growth of *A. muciniphila*. The dependency on GlcNAc or GalNAc is caused by the absence of a gene coding for GlmS, which mediates the aminating reaction from Fru6P to GlcN6P with glutamine as amino donor (Fig. 3), while Amuc‐NagB was shown to be NagB indeed. The absence of GlmS enzyme and the presence of GlcNAc in the ecological niche of *A. muciniphila* resulted in an alternative pathway for peptidoglycan formation.

This finding supported the development of a growth medium that allows the use of *A. muciniphila* in preclinical and phase I clinical trials. Additionally, a minimal medium can be used for detailed physiological studies, and manipulation. With this information, we adjusted an existing genome‐scale metabolic model by deleting the reaction mediated by GlmS. This yielded a more accurate prediction of growth possibilities and growth rate by the metabolic model compared with the previous version of the model. The development of the defined minimal medium for *A. muciniphila* was based on gathered knowledge about this species and contributed to a better understanding of this organism and it evolutionary adaptation to mucin components.

## Experimental procedures

### Culturing *A. muciniphila,* optical density and HPLC

*Akkermansia muciniphila* was grown in anoxic bottles containing 10 ml CP medium as described before without ammonium chloride (Derrien *et al*., 2004), supplemented with 6 g/l of l‐threonine. Initial tests were performed with varying concentrations of GlcNAc (1 mM, 5 mM, and 10 mM) and 50 mM of glucose. Specific growth rates were seen to increase with increasing GlcNAc concentration, and 25 mM sugar was selected for further study, as indicated in Table S1. It should be noted that we used anoxic bottles to culture the bacteria and added the sugars with a syringe and filter after autoclaving. This could yield slightly lower total amounts, but in all cases, the initial sugar concentrations remained above 20 mM sugar, except for GluN where a lower amount of sugar was tested. Inoculum for the determination of growth speed was grown in basal mineral medium supplemented with 6 g/l l‐threonine and 25 mM of GlcNAc. Inoculum for the determination of sugar essentiality was grown on mucus medium as described before (Derrien *et al*., 2004). A volume of 100 μl of cultures in stationary phase was used as inoculum. Growth was determined by measuring the optical density at 600 nm. High‐pressure liquid chromatography (HPLC) was used to determine sugar consumption and SCFA production, as described before (Derrien *et al*., 2004; Ouwerkerk *et al*., 2016; Ottman *et al*., 2017a). Glucosamine (GlcN) concentration was determined by reagent‐free ion chromatography using a Dionex™ ICS‐5000 (Thermo Scientific, Sunnyville, CA, USA), with a Dionex CarboPac PA20 Analytic column and 20 mM NaOH as eluent. Further settings were according to manufacturer's application note. Experiments were performed twice in duplicate; significance of differences was determined by a paired *T*‐test.

### Identification of GlmS and NagB homologues in *A. muciniphila*

*Escherichia coli* K12 NagB (NP_415204.1) and GlmS (NP_418185.1) amino acid sequences were used to identify enzymes that can mediate the formation of GlcN6P from Fru6P in *Akkermansia* (taxid:239934). This was performed by selecting best bidirectional blastp (protein‐protein BLAST) hits between both genomes (Altschul *et al*., 1990). Analysis was performed on February 10, 2017.

### Enzyme expression and purification

The *A. muciniphila* gene NagB on locus Amuc_1822 (AMUC_RS09725) was amplified using the forward primer 5′‐ATAGAGGTACCATGATCGGGGTGGAAAGT‐3′ and the reverse primer 5′‐ TATGCCCTAGGTTA**ATGGTGGTGGTGATGATG**AAGGAGGGAAGCAGCCC‐3′, adding a C‐terminal His‐tag (bold in primer sequence) and restriction sites (underlined in primer sequence). Genomic DNA from *A. muciniphila* ATCC BAA‐835 was used as template. The purified amplicon and vector pCDF‐1b (Novagen, Merck Milipore, Darmstadt, Germany) were digested using enzymes XmaIJ (Thermo Fisher Scientific) and KpnI HF (New England Biolabs) in Tango buffer (Thermo Fisher Scientific) for 1.5 h at 37°C. The DNA was purified again and ligated with 3:1 ratio (insert:vector) using T4 ligase (manufacturers protocol, Thermo Fisher Scientific). Competent *Escherichia coli* DH10B (manufacturers protocol, New England Biolabs) were transformed with 2 μl ligation mixture and plated on LB agar with 50 ug/ml spectinomycin. A selected colony was grown in 10 ml liquid LB until an OD_600_ of 1 after which plasmids were purified. *Escherichia coli* BL21 (DE3) (manufacturers protocol, New England Biolabs) was transformed with 1 μl plasmid containing 60 ug/ml DNA, and 100 μl was plated on LB agar with 50 ug/ml spectinomycin. The transformed strain was grown in 250 ml liquid LB medium with 50 ug/ml spectinomycin until an OD_600_ of 0.5‐1 after which is was induced with 1 mM IPTG for 4 h at 37°C. The bacteria were collected by centrifugation at 5000 *g* for 10 min and suspended in lysis buffer (50 mM Tris‐HCl pH 7.5, 200 mM NaCl, 1 mM MgCl_2_, 5 mM DTT, 1 mM PMSF). The cells were lysed by three passages in a French Press (15 000 psi, Pressure Cell Homogeniser, Stansted Fluid Power Ltd., Essex, UK). The lysate was cleared by centrifugation for 10 min at 16 000 *g*. The proteins were purified using a nickel column (manufacturers protocol, Ni‐NTA Agarose, Qiagen GmbH, Hilden, Germany). Purified fractions were dialyzed against 1 L of buffer A (25 mM KH_2_PO_4_/K_2_HPO_4_, pH 7.0, 0.5 mM PMSF, 1 mM DTT, 1 mM EDTA). Purity of the enzymes was checked using SDS‐PAGE. Enzyme concentration was determined by BCA protein assay (manufacturers protocol, Thermo Fisher Scientific).

### Enzyme assays

Enzyme assays were performed as described before (Kwiatkowska‐Semrau *et al*., 2015). Shortly, 1.5 ml tubes were filled with the buffer and substrates as indicated in Table S3. The enzymes were added last, after which the tubes were vortexed shortly and incubated at 37°C for exactly 3 min. Subsequently, the tubes were placed at 99°C for 1 min and cooled on ice. To acetylate GlcN6P, 100 μl of 10% acetic acid in acetone and 200 μl of a saturated NaHCO_3_ solution were added, after which the samples were incubated for 3 min at 20°C and 3 min at 99°C. The samples were cooled on ice again, after which 200 μl of a 0.8 M potassium tetraborate hexahydrate solution was added. The samples were incubated for 3 min at 99°C and cooled on ice. Finally, 800 μl of this solution was added to 5 ml of reagent A (1 g of 4‐dimethylaminobenzaldehyde in 100 ml of glacial acetic acid to which 1.5 ml 37% HCl is added). The samples were incubated at 37°C for 30 min, after which the absorbance was measured at 585 nm (manufacturer). The Km and Vmax values were determined by plotting the obtained data on a Lineweaver‐Burk plot (Burk *et al*., 1934).

### Genome‐scale constraint‐based metabolic model

The genome‐scale constraint‐based model of *A. muciniphila* AkkMuc_588 reported previously (Ottman *et al*., 2017a) was used to assess growth potential on different media. The core of the model is ***S*** the stoichiometric matrix. ***S*** is an *m *× *n* matrix, where *n* is the number of metabolites and *m* is the number of reactions in the model, 734 and 745 respectively. Entries of ***S*** represent the stoichiometric coefficients of each metabolite in each reaction. In addition to reactions describing metabolic interconversions and transport, the model also contain reactions that describe the media composition and the uptake and secretion potential of the organism, these are the so‐called exchange reactions. The usual convention (followed in the *A. muciniphila* model) is to represent uptake and production as negative and positive fluxes through the exchange reactions respectively. The model was used to simulate steady state as, in such situation, production of intracellular metabolites equals consumption, and the model was used to identify sets of reactions (or pathways) that carry flux in such a case. Moreover, flux balance analysis (FBA) can be used to identify optimal (maximal or minimal) theoretical values for selected objectives such as biomass synthesis or metabolite uptake or production (Orth *et al*., 2010).

Model simulations were performed by simulating the experimentally tested media. Unlimited secretion was allowed for metabolites in the media by setting the upper bounds of the corresponding exchange reactions to 1000. Abundant metabolites in the media such as water, P, Mn, Mg, Fe, Cu, Co, Cl, H_2_O_2_, sulphate and dimethylbenzimidazole, were set to be non‐limiting by setting the lower bounds of the corresponding exchange reactions to −1000. Simulations involving threonine media supplementation were performed with a limited maximal possible uptake of 1. For all the tested sugars, a range of uptake rates between 0 and 10 was tested. Arbitrary units (a.u.) were used in all simulations. Estimations of changes in fluxes in different growth conditions were performed by assessing the variations in the maximum and minimum flux values through each reaction compatible with maximal growth rate. FBA and model simulations were performed using python version 2.7.12 (Python, 2017) the cobrapy library (version 0.4.1) (Ebrahim *et al*., 2013) and gurobi solver (Gurobi Optimizer Version 6.5.1 linux64) (Gurobi, 2016).

## Contributions

All authors read and approved the final manuscript.

## Availability of data and materials

The genome‐scale metabolic model of *Akkermansia muciniphila* AkkMuc_588_v2 is available as supplementary material together with a list of the changes respect to the previous version. Supplementary file contains the AkkMuc_588_v2 model in SBML level 2 and in table (xls) format. The file also contains documentation indicating the changes respect to the previous version. Finally, to ease model reutilization, we have included a Python script to perform growth simulations on different media. The script requires the COBRApy library (and has been tested with version 0.4.1) (Ebrahim *et al*., 2013) and an LP solver such as Gurobi (Gurobi, 2016). This model was deposited in BioModels (Chelliah *et al*., 2015) and assigned the identifier MODEL1710040000.

## Conflict of Interests

The authors declare that they have no competing interests.

## Supporting information


**Fig. S1.** Growth of *A. muciniphila* on CP medium supplemented with 25 mM of each glucose and GlcNAc.
**Fig. S2.** SDS‐PAGE gel of overexpressed protein purification using a Ni‐column.
**Fig. S3.** Growth of *A. muciniphila* on CP medium supplemented with 25 mM GlcNAc, 6 g/l Thr and 0 mM or 50 mM NH_4_Cl.
**Table S1.** Composition of tested media.
**Table S2**. Determination of protein concentrations by BCA assay used for enzyme assays.
**Table S3.** Overview of substrates used in enzyme assay.
**Table S4**. BlastP of *Escherichia coli* K12 NagB (NP_415204.1) against *Akkermansia* (taxid:239934).
**Table S5.** BlastP of *A muciniphila* NagB (CDB55261.1) against all sequence except *Akkermansia* (taxid:239934).
**Table S6.** BlastP of *Escherichia coli* K12 GlmS (NP_418185.1) against *Akkermansia* (taxid:239934).
**Table S7.** BlastP of *Verrucromicrobium spinosum* GlmS (WP_009962724.1) against *Akkermansia muciniphila*.
**Table S8.** Control reactions for enzyme assay of *A. muciniphila* NagB.
