# Supplementary material for: Model‐driven design of a minimal medium for Akkermansia muciniphila confirms mucus adaptation
Source: Microb Biotechnol. 2018 Jan 26;11(3):476–85. doi: 10.1111/1751-7915.13033 (PMC5902328; doi:10.1111/1751-7915.13033)
Supplement: Supplementary file 1 — Fig. S1. Growth of A. muciniphila on CP medium supplemented with 25 mM of each glucose and GlcNAc. Fig. S2. SDS‐PAGE gel of overexpressed protein purification using a Ni‐column. Fig. S3. Growth of A. muciniphila on CP medium supplemented with 25 mM GlcNAc, 6 g/l Thr and 0 mM or 50 mM NH4Cl. Table S1. Composition of tested media. Table S2. Determination of protein concentrations by BCA assay used for enzyme assays. Table S3. Overview of substrates used in enzyme assay. Table S4. BlastP of Escherichia coli K12 NagB (NP_415204.1) against Akkermansia (taxid:239934). Table S5. BlastP of A muciniphila NagB (CDB55261.1) against all sequence except Akkermansia (taxid:239934). Table S6. BlastP of Escherichia coli K12 GlmS (NP_418185.1) against Akkermansia (taxid:239934). Table S7. BlastP of Verrucromicrobium spinosum GlmS (WP_009962724.1) against Akkermansia muciniphila. Table S8. Control reactions for enzyme assay of A. muciniphila NagB. [file MBT2-11-476-s001.docx]

**Supplementary Figures and Tables**


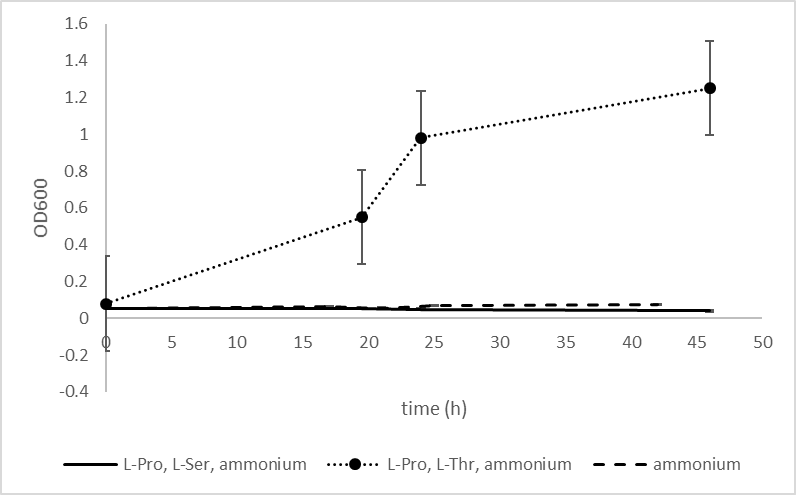


Figure S1 Growth of *A. muciniphila* on CP medium supplemented with 25 mM of each glucose and GlcNAc. As nitrogen source was added 4 g/L of L-proline and either L-serine or L-threonine. In all bottles 0.3 g/L NH_4_Cl was added. Growth was only observed with the addition of L-threonine.


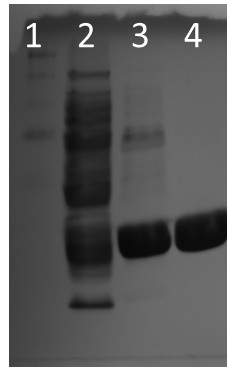


Figure S2 SDS-PAGE gel of overexpressed protein purification using a Ni-column. In lane 1: marker, lane 2: *E. coli* BL21-Amuc_1822 CFE, lane 3: wash flow through, lane 4: purified protein amuc_1822.


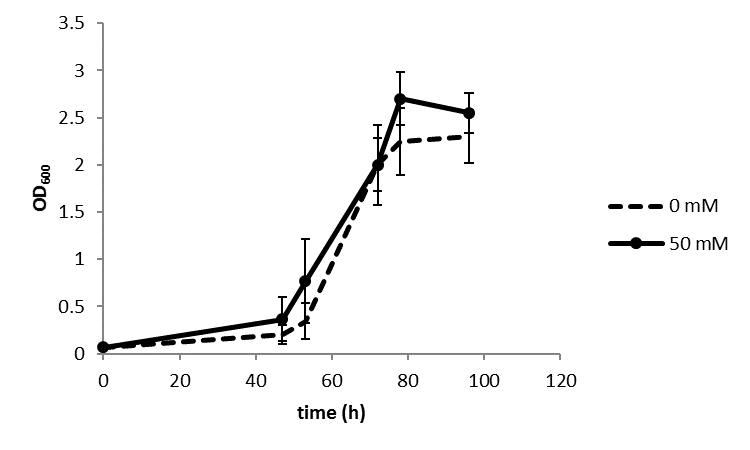


Figure S3 Growth of *A. muciniphila* on CP medium supplemented with 25mM GlcNAc, 6g/L Thr and 0mM or 50mM NH_4_Cl.

Table S1 Composition of tested media. The indicated compounds are added to CP medium without ammonium (Derrien, et al., 2004).

| Carbon source | | |
| --- | --- | --- |
| Glucose (mM) | GlcNAc (mM) | Other sugars (mM) |
| 12.5 | 12.5 |  |
| 0 | 0 |  |
| 0 | 25 |  |
| 12.5 | 12.5 |  |
| 0 | 0 |  |
| 0 | 25 |  |
| 12.5 | 0 | 12.5 GlcN |
| 0 | 0 | 25 GalNAc |
| 0 | 0 | 25 GlcN |
| 12.5 | 0 | 12.5 GlcN |
| 0 | 0 | 25 Fru |

Table S2 Determination of protein concentrations by BCA assay used for enzyme assays. The BSA was used for a standard curve. The enzyme was diluted 5 times and 40 times to determine the protein concentration.

| BSA (ug/mL) | 590nm | 450nm | OD590/450 | |
| --- | --- | --- | --- | --- |
| 0 | 0.45 | 0.74 | 0.61 |  |
| 1 | 0.46 | 0.75 | 0.61 |  |
| 5 | 0.50 | 0.73 | 0.69 |  |
| 10 | 0.57 | 0.70 | 0.81 |  |
| 50 | 0.97 | 0.54 | 1.78 |  |
| 100 | 1.31 | 0.42 | 3.14 |  |
|  |  |  |  |  |
| 5 × diluted Amuc-NagB | 1.82 | 0.29 | 6.28 |  |
| 40× diluted Amuc-NagB | 0.78 | 0.62 | 1.25 |  |
|  |  |  |  |  |
| Concentration Amuc-NagB (mg/mL) | | | SD |  |
|  | 1.10 |  | 0.04 |  |

Table S3 Overview of substrates used in enzyme assay.

|  |  | Substrates (mM) | | | Activatorᶧ (mM) | Enzyme (µL) |
| --- | --- | --- | --- | --- | --- | --- |
|  | # tube | Fru6P | NH4 | GlcN6P | (GlcNAC-6P) |  |
| AMINATING | 1 | 15 | 50 |  | 0.25 | 5 |
|  | 2 | 10 | 50 |  | 0.25 | 5 |
|  | 3 | 7.5 | 50 |  | 0.25 | 5 |
|  | 4 | 5 | 50 |  | 0.25 | 5 |
|  | 5 | 2 | 50 |  | 0.25 | 5 |
|  | 6 | 1 | 50 |  | 0.25 | 5 |
|  |  |  |  |  |  |  |
|  | 7 | 20 | 50 |  | 0.25 | 5 |
|  | 8 | 20 | 25 |  | 0.25 | 5 |
|  | 9 | 20 | 10 |  | 0.25 | 5 |
|  | 10 | 20 | 5 |  | 0.25 | 5 |
|  | 11 | 20 | 2.5 |  | 0.25 | 5 |
|  |  |  |  |  |  |  |
| DEAMINATING | 12* |  |  | 15 | 0.25 | 1 |
|  | 13* |  |  | 10 | 0.25 | 1 |
|  | 14* |  |  | 7.5 | 0.25 | 1 |
|  | 15 |  |  | 5 | 0.25 | 1 |
|  | 16 |  |  | 2 | 0.25 | 1 |
|  | 17 |  |  | 1 | 0.25 | 1 |
|  |  |  |  |  |  |  |
| Standard curve | 18 |  |  | 0 |  |  |
|  | 19 |  |  | 0.5 |  |  |
|  | 20 |  |  | 1 |  |  |
|  | 21 |  |  | 2 |  |  |
| Control GlcNAC6P |  |  |  |  |  |  |
|  | 22 |  |  |  | 0.25 |  |

ᶧ Added in 2 out of 5 replicates. * Not used in determination of Km.

Table S4 BlastP of *Escherichia coli* K12 NagB (NP_415204.1) against *Akkermansia* (taxid:239934). All hits are shown.

| Description | Max score | Total score | Query cover | E value | Ident | Accession |
| --- | --- | --- | --- | --- | --- | --- |
| glucosamine-6-phosphate deaminase [Akkermansia muciniphila CAG:154] | 177 | 177 | 92% | 5.00E-55 | 40% | CDB55261.1 |
| MULTISPECIES: glucosamine-6-phosphate deaminase [*Akkermansia*] | 176 | 176 | 92% | 8.00E-55 | 40% | WP_067570388.1 |
| glucosamine-6-phosphate deaminase [*Akkermansia glycaniphila*] | 175 | 175 | 87% | 2.00E-54 | 42% | WP_067775984.1 |
| glucosamine-6-phosphate deaminase [*Akkermansia muciniphila*] | 175 | 175 | 90% | 1.00E-53 | 41% | WP_065529150.1 |
| glucosamine-6-phosphate deaminase [*Akkermansia muciniphila*] | 175 | 175 | 90% | 1.00E-53 | 41% | WP_012420854.1 |
| glucosamine-6-phosphate deaminase [*Akkermansia sp.* CAG:344] | 172 | 172 | 92% | 3.00E-53 | 39% | CDD98135.1 |
| glucosamine-6-phosphate deaminase [*Akkermansia muciniphila*] | 173 | 173 | 90% | 5.00E-53 | 40% | WP_031931289.1 |
| hypothetical protein HMPREF3038_03217 [*Akkermansia* sp. KLE1797] | 24.6 | 24.6 | 4% | 7.8 | 73% | KXT46402.1 |

Table S5 BlastP of *A muciniphila* NagB (CDB55261.1) against all sequence except *Akkermansia* (taxid:239934). Top 5 hits are shown.

| Description | Max score | Total score | Query cover | E value | Ident | Accession |
| --- | --- | --- | --- | --- | --- | --- |
| glucosamine-6-phosphate deaminase [*Rubritalea squalenifaciens* DSM 18772] | 354 | 354 | 82% | 3.00E-120 | 67% | SHJ43774.1 |
| glucosamine-6-phosphate deaminase [*Rubritalea marina*] | 319 | 319 | 81% | 2.00E-106 | 61% | WP_018969388.1 |
| glucosamine-6-phosphate deaminase [*Capnocytophaga canis*] | 317 | 317 | 79% | 2.00E-100 | 60% | WP_042008125.1 |
| Glucosamine-6-phosphate deaminase [*Flavobacterium aquidurense*] | 305 | 305 | 80% | 2.00E-99 | 56% | KQB39432.1 |
| glucosamine-6-phosphate deaminase [*Capnocytophaga canis*] | 314 | 314 | 79% | 2.00E-99 | 59% | WP_042347886.1 |

Table S6 BlastP of *Escherichia coli* K12 GlmS (NP_418185.1) against *Akkermansia* (taxid:239934). All hits are shown.

| Description | Max score | Total score | Query cover | E value | Ident | Accession |
| --- | --- | --- | --- | --- | --- | --- |
| von Willebrand factor type A domain protein [*Akkermansia* sp. KLE1797] | 32 | 32 | 17% | 0.68 | 26% | KXT50996.1 |
| MULTISPECIES: hypothetical protein [*Akkermansia*] | 32 | 32 | 17% | 0.71 | 26% | WP_067570323.1 |
| queuine tRNA-ribosyltransferase [*Akkermansia muciniphila* CAG:154] | 29.3 | 29.3 | 4% | 3.9 | 40% | CDB55386.1 |
| tRNA guanosine(34) transglycosylase Tgt [*Akkermansia* sp. 54_46] | 29.3 | 29.3 | 4% | 4.2 | 40% | OLA88015.1 |
| tRNA-guanine(34) transglycosylase [*Akkermansia muciniphila*] | 29.3 | 29.3 | 4% | 4.2 | 40% | WP_031930139.1 |
| tRNA guanosine(34) transglycosylase Tgt [*Akkermansia muciniphila*] | 29.3 | 29.3 | 4% | 4.4 | 40% | WP_012419333.1 |

Table S7 BlastP of *Verrucromicrobium spinosum* GlmS (WP_009962724.1) against *Akkermansia* *muciniphila*. All hits are shown.

| Description | Max score | Total score | Query cover | E value | Ident | Accession |
| --- | --- | --- | --- | --- | --- | --- |
| tRNA-guanine(34) transglycosylase [*Akkermansia muciniphila*] | 30 | 30 | 5% | 0.83 | 42% | WP_031930139.1 |
| tRNA guanosine(34) transglycosylase Tgt [*Akkermansia muciniphila*] | 30 | 30 | 5% | 0.84 | 42% | WP_012419333.1 |
| hypothetical protein [*Akkermansia muciniphila*] | 27.3 | 27.3 | 11% | 6 | 25% | WP_065529387.1 |

Table S8 Control reactions for enzyme assay of *A. muciniphila* NagB. The amount of GlcN6P formed is not above 0.01 mM in 10 minutes.

| Content assay | OD_585_ | GlcN6P (mM) |
| --- | --- | --- |
| 10 mM Fru6P + 10mM glutamine  5 uL Enzyme | 0.006 | 0.01 |
| 50 mM NH4Cl + 7,5mM Fru6P | 0.000 | 0.00 |
| 5 uL Enzyme | 0.000 | 0.00 |
